# Correspondence in reference to the previously published Epub manuscript: “Murt Ahmet et al. Hepatitis B reactivation in hematopoietic stem cell transplanted patients: 20 years of experience of a single center from a middle endemic country. *Annals of Hematology* 2020; 99: 2671-2677”

**DOI:** 10.1007/s00277-021-04436-9

**Published:** 2021-02-01

**Authors:** Marco Picardi, Claudia Giordano, Della Pepa Roberta, Novella Pugliese, Aldo Leone, Giuseppe Gentile, Fabrizio Pane

**Affiliations:** 1 Department of Clinical Medicine and Surgery, Hematology Unit, Federico II University Medical School, Via Sergio Pansini, 5, 80131 Naples, Italy; 2 Department of Translational and Precision Medicine Sapienza Università di Roma, Rome, Italy

Dear Editor,

The increased morbidity and mortality due to hepatitis B virus (HBV)-related adverse events following hematopoietic stem cell transplantation (HSCT) have raised questions regarding its efficacy and widespread adoption especially in areas with intermediate and high levels of endemicity for HBV infection [1, 2].

We read with great interest Ahmet et al. study published ahead of print on July 31, 2020, in *Annals of Hematology* [3]. This retrospective study assessed the incidence of HBV reactivation in a series of 15 hepatitis B surface antigen (HBsAg)-seropositive adult patients receiving HSCT for hematological malignancies (cellular lymphoplasm cancers, 12 cases; acute leukemia, 3 cases) between 1994 and 2015 at the endemic area of Istanbul (Turkey). As prophylaxis against HBV reactivation, all patients received lamivudine from HSCT until 6 months after transplant, according to local guidelines. After a median of 9 months (range, 2–24 months) post-HSCT, five out of 15 patients developed HBV reactivation or exacerbation of HBV replication which led to hepatic impairment with death in 3 cases, thus experiencing at least 20% of episodes of fulminant and fatal acute hepatitis due to HBV infection. Overall survival (OS) projected at 60 months was 60%. The authors underscored that HBV prophylaxis extended over 1 year should be given for HBsAg-positive patients undergoing HSCT for hematological malignancies, regardless of the type of drug against HBV prescribed.

**Fig. 1:**
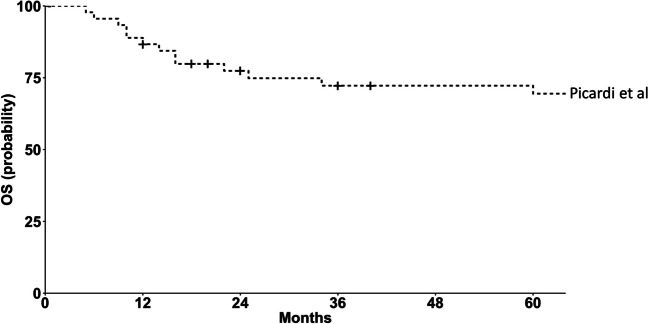
Kaplan-Meier curve showing overall survival (OS) of the HBsAg-positive patients (*n* = 45, including 5 patients undergoing hematopoietic stem cell transplantation owing to refractory or relapsed disease) homogeneously and systematically protected with tenofovir disoproxil fumarate (from front-line immunochemotherapy until 1 year after immunosuppressive treatment discontinuation) in the present study (Picardi et al.)

Several attempts have been made in this setting of patients to define the best antiviral strategy [4, 5]. There is the need for interventions that could mitigate HBV infectious morbidity and mortality (especially primary antiviral prophylaxis [PAVP] with a new-generation oral nucleotide analog [ONA] with a strong antiviral activity and high genetic barrier to resistance) to establish the full value of HSCT in patients with hematological malignancies and HBsAg positivity [6]. We reported in *Blood* of 2019 [7] preliminary encouraging single-center efficacy and safety results of a prospective trial in HBsAg-seropositive patients receiving homogeneous PAVP with only one type (for all patients) of ONA concurrently with immunochemotherapy as remission induction for diffuse large B cell lymphoma (DLBCL). Herein, we describe the mature results from this trial, in terms of the increasing number of patients with a longer follow-up. During the 2009–2020 period at the Hematology Unit of the Federico II University of Naples in southern Italy (endemic area for HBV infection), a real-life consecutive series of 45 HBsAg-seropositive (HBV DNA serum levels detectable in 35% of cases [median, 1000 IU/mL]) patients (median age, 53 years) with DLBCL received tenofovir disoproxil fumarate (TDF; 245 mg orally daily) beginning 1 week before R-CHOP-21 [8] and scheduled to continue for 12 months after completion of immunosuppressive treatments. The clinical presentations were aggressive (advanced-stage disease in 100% of patients, IPI-scores ≥ 3 in 75% of patients), thus requiring 6 cycles of R-CHOP-21 plus 2 additional courses of rituximab. Thereafter, 5/45 (11%) patients received HSCT for primary refractory or early relapsed DLBCL. The antiviral prophylactic drug was well tolerated in all patients with no discontinuations for any adverse event, toxicity, or non-compliance (including five transplanted patients, who received TDF from R-CHOP-21 until 1 year following transplant). After a median follow-up of 80 months, no emergent HBV DNA or exacerbation of HBV replication was registered in any of the 45 patients, in addition, none of the patients developed acute hepatitis. Six-year OS was 71% (95% CI, 57%–85%), as shown in Fig. 1. With the systematic, prompt, and sustained use of PAVP with TDF (until 1 year from immunosuppressive treatment discontinuation), we were able to drastically bring down the rate of HBV-related adverse events in patients with lymphoproliferative malignancies in advanced-stage receiving repeated R-CHOP-21 courses and subsequently HSCT (if needed), complying fully with the dose-dense and dose-intensity of the anti-cancer strategy employed thus leading to durable long-term efficacy and consequently improving outcome.

Guided and encouraged by the evidence of a high level of efficacy provided by the TDF investigators, we launched a prospective multicenter clinical trial in Italy in patients with DLBCL and HBsAg positivity treated front-line with R-CHOP-21 and tenofovir alafenamide (EudraCT number 2019–000159-14; ClinicalTrials.gov ID NCT03804372).

[Human studies have been approved by the appropriate ethics committee and have therefore been performed in accordance with the ethical standards laid down in the 1964 Declaration of Helsinki and its later amendments. All patients in our study gave their informed consent prior to their inclusion in the study].
